# Hygiene and Eating Healthy Habits and Practices in Spanish Families with Children Aged 6 to 14

**DOI:** 10.3390/ijerph17228671

**Published:** 2020-11-22

**Authors:** Petra María Pérez Alonso-Geta, M. Carmen Bellver Moreno

**Affiliations:** Department of Education Theory, University of Valencia, 46010 Valencia, Spain; petra.m.perez@uv.es

**Keywords:** eating, family typology, hygiene habits, childhood, adolescence

## Abstract

During childhood and pre-adolescence, the family environment is key to initiating and consolidating healthy styles in children through a balanced diet and basic hygiene habits. This study analyses hygiene, nutrition and health practices in Spanish families with children between 6 and 14 years of age according to the type of family (nuclear, single-parent or reconstituted) and the quantity, age and gender of the children. A representative Spanish national sample of 1103 Spanish parents, 270 fathers and 833 mothers, with children aged 6 to 14, is analysed. The study is descriptive, using statistical techniques with classic indicators (means, percentages). The results show that nuclear families manifest healthier habits, in general, and consider the consumption of pastries, ultra-processed food and excessive amounts of salt to be harmful. Furthermore, this family typology develops healthy and hygienic habits, such as brushing teeth daily, sleeping at least 8 h a day, drinking a glass of milk a day, eating fish more than once a week and eating fast food sporadically. They are also concerned about their children’s annual medical check-ups (paediatrician and dentist). It is concluded that the family type is related to the hygiene and feeding habits of the children.

## 1. Introduction

Lifestyles have a profound impact on the lives of individuals. A lifestyle encompasses the habits and behaviours that a person develop throughout their life and which are especially influenced by their family, their school and their community, collectively shaping their personality [1]. Healthy lifestyles are a key indicator of personal well-being and are closely related to a balanced diet, an appropriate body weight, regular physical activity and no use of alcohol or tobacco, among others [2]. Children and preteens (6–14 years old) are at a key stage as they acquire and maintain eating, hygiene and physical exercise healthy habits which will remain throughout their lives [3,4].

A healthy diet requires specific nutrition adapted to the individual’s stage of life, gender and physical activity. This means ensuring the presence, balance and moderation of all nutrients [5,6]: macronutrients such as carbohydrates, proteins and fats, as well as micronutrients such as vitamins, minerals and water. In addition, it is necessary to increase the variety of sources, both animal and plant-based, the organism needs to function properly. Among all developmental stages, childhood and puberty (6–14 years old) are considered to be stages of slow, but steady, physical growth [7]. Therefore, during this period, children should eat fruit, vegetables, carbohydrates, dairy products, meat, fish, eggs, legumes and nuts to provide an adequate supply of the calories and nutrients they need. They should also reduce the amount of industrially produced bakery products (sweet buns, biscuits, cakes, etc.) and ultra-processed products (cold meats, frozen pizzas, sweets, etc.), which are characterised by their high calorie content in the form of fats and sugars.

Besides, adolescence is a crucial stage to maintain and strengthen essential hygiene and eating habits that will be part of a subject during their adult life [8]. Breakfast is an example, as it has been shown that poor breakfast habits can be related to metabolic problems in adulthood [9]. Moreover, this stage can be prone to develop risk factors [10] that could result in eating disorders, such as skipping a meal, binge eating, excessive consumption of fast food, excessive snacking or low-calorie diets, all of which could result in higher health risks.

Studies suggest that family and school influence children’s eating behaviours through modelling performed in educational and childcare settings [11,12]. The school should restrict the sale of sweetened beverages in their premises and adopt healthy eating and hygiene practices. According to parents and teachers in secondary schools, easy access to carbohydrate-rich, yet nutrient-poor, foods together with limited access to nutritious foods, because of their high price, are risk factors in the school environment [13]. In addition, specific food policies in this area should be strengthened.

Therefore, analysing eating and hygiene habits within the family environment is crucial to prevent future problems in children as, for example, obesity. Currently, obesity is a major health problem in our country; 19% of obesity in children is related to parents’ eating habits [14] as well as to chronic disease and mental illness [15].

The family is the first context in which these healthy habits are implemented and developed [16]. Ref [17] emphasizes that the lessons learned in the family setting leave an imprint that will accompany human beings throughout their lives, particularly with regards to health. In our country, families have undergone a profound transformation. In recent decades, new typologies of families have appeared (single-parent and reconstituted), leading to new family structures with new ways of relating to one another.

Along with the nuclear family (father, mother and children), which is still the predominant one in our country, the single-parent family (father, or mother, and children) [18] is on the increase. Reconstituted families, consisting of a couple with non-common children from previous relationships, are also on the rise. These changes in family typology have introduced important changes in the roles that families have traditionally played in raising their children, directly affecting the eating, hygiene and health practices and habits they develop [19].

Food and hygiene must be adapted to all stages of the subject’s development. Childhood and adolescence are, however, the most favourable stages to develop healthy habits and practices within the different areas of socialization: family, school and community [20]. It is therefore necessary, during this stage (6 to 14 years old), to continue promoting within the family environment hygiene habits initiated in early childhood, such as daily showers, tooth brushing or changing clothes daily. They will be fundamental for their future consolidation during adolescence and adulthood. [17,21,22,23].

In addition, basic eating habits developed during this stage should include a balanced diet, physical exercise [24], moderate salt and sugar intake, eating vegetables several times a week, avoiding excessive use of ultra-processed foods or baked goods, limiting soft drinks and promoting the daily intake of fruit and dairy products. All of these are healthy guidelines that will accompany them throughout adulthood. It is also important to eat together as a family and not in front of screens (computers, consoles, etc.) and to limit advertising from certain media, from fashion, etc. [25,26].

The aim of this study is to provide a descriptive analysis of the habits and practices regarding hygiene, food and health that exist in Spanish families with children aged 6 to 14, according to their family structure (nuclear, single-parent or reconstituted), the number of children per household, their age and their gender. We highlight the importance that this insight can bring to policy-making and to creating appropriate guidelines for a healthy lifestyle during the period under study.

## 2. Materials and Methods

### 2.1. Participants

The representative sample consisted of 1103 Spanish parents; 270 fathers (24.5%) and 833 mothers (75.5%) with children from 6 to 14 years old (M = 10.21; SD = 2.65). The distribution by gender was 486 girls (48.6%) and 514 boys (51.4%). According to the school they attended, 72.5% were enrolled in public schools, 21.6% in charter schools and 5.9% in private schools (Table 1).

A random sampling was conducted establishing quotas independent of gender, age and autonomous region. *N* = 1000 with a sampling error of 3.2% for a 95.5% confidence level.

Table 2 shows the family profile of the sample interviewed according to their marital status (single, married, partnership, separated or widowed) and the children’s parentage.

### 2.2. Measuring Instrument Questionnaire

The data collection required a quantitative methodology developed through the following steps: (a) Elaboration of the measuring instrument: questionnaire; (b) elaboration of the technical sheet: population characteristics and sampling; (c) field work: application, time and application of the questionnaire to the sample; and (d) statistical treatment of the data.

Initially, and based on the objectives of this work, group dynamics were conducted with children and teachers from the segment studied (6–14 years old). This made it possible to outline a structured questionnaire—the data collection instrument for this study. The variables and units of observation were structured around the different objectives of the research.

The final questionnaire consisted of 29 questions on education and family interaction patterns (consumption, educational and family interaction guidelines) in households with children aged 6 to 14, with a Likert type scale (never = 1, only when necessary = 2, once a week = 3, several times a week = 4, daily = 5). The methodology used in this study consisted of 25 to 30-min personal interviews to parents with children aged 6 to 14 in their households.

In order to detect the adequacy of the questionnaire to the objectives of the study, the elaboration and subsequent finalization of the questionnaire followed the corresponding pilot survey—standard completion of 5% of the surveys to parents—conducted in the different types of schools analysed (public, charter and private) in the city of Valencia.

### 2.3. Data Analysis

In the analysis of the results, statistical techniques have been used with classic indicators (means, percentages, etc.) to allow a descriptive analysis, using the SPSS v.23 programme (IBM, Madrid, Spain).

### 2.4. Ethical Considerations

The ethical approval for this study, carried out by the company EMER-EGFK, is in accordance with the standards of the ESOMAR code of conduct. In addition, the entire research process adhered to the ethical principles for research with people, outlined in the Declaration of Helsinki [27] which guarantees the principles of respect for people, beneficence and justice. Participants privacy and confidentiality were preserved and protected. All participant data were made anonymous and all possible identifiers were removed. All the information from this study is password protected.

## 3. Results

### 3.1. Parental Assessment of the Eating Habits of Children Aged 6 to 14 according to Household Profile (Nuclear, Reconstituted, Single Parent), Number of Children, Their Age and Their Gender

The analysis of eating habits in families with children aged 6 to 14 requires the opinion and assessment of parents with children of this age group, regarding essential eating habits: consumption of industrial baked goods (buns, cakes, biscuits, chocolates) as unhealthy ultra-processed foods; presence of fibre in their diets; salt and candy consumption; regular consumption of vegetables during meals or replacing them with other foods they like more; consumption of soft drinks as they are wrongly considered to provide energy and/or considering that vitamins can replace daily fruit consumption. Table 3 shows the percentage and means of eating practices and habits in families with children aged 6–14 according to the household profile (nuclear, reconstituted, single parent).

In relation to the differences in opinions and eating habits between the various groups analysed, no significant differences are observed among household profiles (nuclear, reconstituted or single parent) with regard to the healthy eating habits such as taking fibre or not using soft drinks or salt excessively.

However, we do find that nuclear families have the lowest rates in unhealthy behaviours, especially the consumption of baked (12%) and ultra-processed goods, considering that salt is not harmful (10%), replacing daily fruit consumption with vitamins (4%) and believing that ‘if a child is chubby, it means that they are healthy’ (5%). The nuclear family is also the one with the lowest percentage (18%) in terms of consenting to give another substitute food if vegetables are not to the children’s liking.

In the case of single-parent families, 35% consider that ‘it is okay if they eat candy now and then’ and 8% consider that it is no longer necessary to monitor their diet as they are no longer babies.

On the opposite extreme, reconstituted families show the highest rates in relation to negative healthy habits: 14% of parents in this group give their children industrial baked goods because that is what they like, 19% give them other types of food if they do not like vegetables, 12% consider that soft drinks are good because they provide energy and, finally, 9% consider that ‘if a child is chubby, they are healthy’.

When looking at the number of children, parents with several children are significantly more in agreement with the statement ‘it is okay if they eat candy now and then’ (34%), which can lead to a greater intake of sweets in their children’s diet. In addition, the least chosen option as a whole, ‘fruit is not necessary if I give them vitamins’, is more accepted among parents with only one child (5%).

If we take into account the children’s age, there are two issues that vary significantly as children reach the age of 11: (a) Parents are less in agreement with the statement ‘if a child is chubby, it means they are healthy’, which can be related to the image models imposed when children reach adolescence, and (b) parents more strongly agree on the statement ‘since they are no longer a baby, it is not important to control their diet’, which confirms the idea that, as children reach adolescence, parents supervise their food and diet less.

In relation to gender, there is a significant difference in the statement ‘if a child is chubby, it means they are healthy’. If the child is male, it has a greater acceptance among parents (7%) than if the child is female (4%).

### 3.2. Parental Assessment of Basic Hygiene and Healthy Eating Habits of Children Aged 6 to 14 according to Household Profile (Nuclear, Reconstituted, Single Parent), Number of Children, Their Age and Their Gender

It is necessary to analyse hygiene habits (daily shower, tooth brushing, sleeping at least 8 h a day, etc.) because, if these habits have been implemented in the age range analysed in this study, they will strengthen in adolescence and adulthood. It is also important to analyse the consumption of fast food and milk (one glass a day) and, regarding health monitoring, the health check-ups by paediatricians and dentists (Table 4).

In the analysis carried out regarding the household profile, it is evident that parents from nuclear households make more of an effort to make sure their children sleep at least 8 h a day (93%) and go to the dentist at least once a year (81%). In addition, in terms of nutrition, nuclear households have the best habits; they significantly have more of a habit of drinking a glass of milk a day (87%) and eating more fish (65%) and are also less likely to go to fast food restaurants (9%). Single-parent families, in second place, and reconstituted families, in third, present lower rates in all these items. Still, most of the three types of families analysed have established basic hygiene habits in their children, such as brushing their teeth, taking a daily shower and sleeping at least 8 h a day.

In addition, the results show that it is significantly more common to go to the dentist at least once a year (82%) in households with several children. When looking at the age group, it is significantly less important for parents of children aged 12–14 to have regular health check-ups with the paediatrician (81%), which illustrates that the importance of health monitoring is mitigated in the pre-adolescence stage.

We find significant differences as children grow older. For example, the habit of drinking a glass of milk a day decreases; children from the 6 to 8 age group usually eat more fish, which declines as they get older; and 12- to 14-year-olds significantly eat more fast food.

No significant differences were observed in terms of the children’s gender.

## 4. Discussion

In our study, the fundamental role that parents have on their children’s eating habits is evident, since they decide on the selection of food, as well as on the quantity and quality provided during childhood and adolescence, as highlighted in several studies [28,29,30].

The period of childhood and puberty is critical to the children’s optimal development [7,24] and families should provide a rich and varied diet of protein, fruits, vegetables and carbohydrates as well as limit the consumption of ultra-processed foods. Our data show that nuclear families have the most appropriate health promotion profile for their children, since they show the lowest consumption of pastries and ultra-processed foods, provide more fresh fruit and vegetables and avoid excessively salty foods, in line with other studies [31].

On the other hand, reconstituted families exhibit more harmful eating habits. They are more in favour of consuming pizza (ultra-processed food), are more permissive with the consumption of sugar (in the form of pastries and sweetened soft drinks) and are more indulgent in regularly substituting vegetables for other foods that are more enjoyable for their children, which can lead to greater food neophobia towards vegetables [32]. All of these harmful family eating habits could multiply the risk of obesity in children if they are associated with low household income [32].

Parents with several children believe that the occasional consumption of sweets is not a risk, indicating a greater acceptance of sweets in their children’s diet [23]. In the case of parents with an only child, the belief that vitamins can be a substitute for fresh fruit consumption is noteworthy.

Regarding the children’s gender, there is a greater acceptance of the statement ‘if a child is chubby, it means it is healthy’ in parents with sons rather than daughters, driven by social pressure on the image of women [22].

Additionally, the results show that, as children grow older, parents reduce supervision of their children’s diet, which can lead to the development of harmful eating habits in teenagers and potentially lead to eating disorders: skipping meals or binge eating, high consumption of fast food [10] and excessive consumption of snacks between meals [33]. Parents’ concern for their children’s physical image also increases with age; from the age of 11, parents do not consider an overweight child is a synonym for health, perhaps influenced by social aesthetic norms. However, as previously discussed, this does not translate into a greater concern and supervision of their children’s diet.

All of this may result in significant changes in the eating habits of Spanish families, particularly the low consumption of dairy products and fish and the increase in ultra-processed foods and sugars, as is also evident in other Latin American countries [1]. These eating habits require to place the focus on improving the diet of families with children aged 6 to 10 to prevent diseases, such as childhood obesity, and to develop activities for the prevention, management and control of other health problems in adulthood [28].

Finally, the different family typologies analysed in our study have developed in their children basic hygiene habits such as brushing their teeth, taking a daily shower and sleeping at least 8 h a day. These guidelines are essential as a measure to avoid the increase of dental caries in school-aged children [30].

## 5. Conclusions

Nuclear families (father, mother, sons and daughters) stand out as the family typology with the best information on healthy eating habits. They have a negative view on the consumption of baked and ultra-processed goods and salt; they also consider vitamins do not replace daily fruit intake and they do not accept that a child’s overweight is a sign of health. In addition, they develop healthy habits in their children, such as sleeping at least 8 h a day, drinking a glass of milk a day, eating fish more than once a week and going to fast food restaurants only when necessary. They are also the most concerned ones about their children’s health check-ups with the paediatrician and the dentist.

Another conclusion from our study is that, as children become older, parents tend to supervise their diet and nutrition less, increasing the risk from the age of 10–12. In addition, it has been ascertained that the 12 to 14 age group considerably increases the consumption of fast, or not healthy, food. Finally, the data obtained show that parents with more than one child are more likely to let their children eat candy from time to time and parents with only one child consider that vitamins can be a substitute for daily fruit consumption.

In order to improve the hygiene and eating habits of children aged 6 to 14, it is necessary that health organizations implement family health policies to develop healthy habits that should be built from within the family: food, physical activity and hygiene. This way, children between the ages of 6 and 14 could be fed adequately and poor nutrition and chronic diseases, such as obesity, could be prevented [34].

Some of the practices that could be developed by means of parental training on health education within the family context include [35]: (a) Choose to eat at home and, in the case of eating out, request healthy menus; (b) do not compulsively buy offers that double the portions, weight or volume of food; (c) avoid eating in front of screens (television, video consoles, computer, etc.), as people tend to eat more or less; (d) read food labelling, especially in the case of ultra-processed foods; (e) learn to buy healthy food responsibly; (f) avoid eating chips, packaged products or snacks, or using dressings; and, (g) avoid the excessive use of salt and sugar, especially in drinks and sugary soft drinks.

This line of action should be supplemented by the implementation of school health education programs on nutrition, hygiene and healthy lifestyle in primary schools (8- to 12-year-olds) and in the first cycle of compulsory secondary education (12- to 14-year-olds) with the aim of educating children about health and healthy lifestyles [36]. These programs should necessarily be in conjunction with community programmes that work with families on the importance of eating habits and physical activity [37].

Limitations of the Study.

Finally, despite the limitations of our study, the conclusions are relevant to continue research in the presented line, delving into the influence of the family in the development of eating habits throughout the child’s development, especially during their first years [29], since these habits will accompany them throughout their lives.

## Figures and Tables

**Table 1:** Distribution of the sample according to age and gender.

Gender/Age	Population	Percentage	Sampling Size	Sampling Error
Total	3,647,400	100%	1000	3.2%
**Gender**				
Males	1,873,903	51.4%	514	4.4%
Females	1,773,497	48.6%	486	4.5%
**Age Group**				
6 to 8 years old	1,138,702	31.2%	312	5.7%
9 to 11 years old	1,223,035	33.5%	335	5.5%
12 to 14 years old	1,285,663	35.2%	352	5.3%

**Table 2:** Profile of the sample interviewed: family typology and parents’ marital status.

Family Tipology	Total	Single	Married	Partnership	Separated or Divorced	Widowed
Total	1103	38	858	95	103	9
Couple with all children in common	900	-	827	67	5	1
Couple with at least one child not in common	58	-	31	27	-	-
Same sex couple with children	1	-	-	1	-	-
Mother with children	133	38	-	-	87	8
Father with children	11	-	-	-	11	-

**Table 3:** Percentage and averages of eating habits in families with children aged 6 to 14 according to household profile (nuclear, reconstituted, single parent), number of children, their age and their gender.

Eating Habits	Total		Home Profile						Number of Children				Children’s Age						Children’s Gender			
	*n* = 1103		Nuclear *n* = 900		Reconstructed *n* = 59		Single Parent *n* = 144		Only CHILD *n* = 417		2 or More *n* = 686		6–8 *n* = 394		9–11 *n* = 351		12–14 *n* = 358		Boy *n* = 586		Girl *n* = 517	
	%	X	%	X	%	X	%	X	%	X	%	X	%	X	%	X	%	X	%	X	%	X
I make sure to include fibre in my children’s diet	71	3.9	70	3.8	75	3.9	76	3.9	71	3.9	71	3.9	73	3.9	71	3.9	69	3.8	72	3.9	70	3.8
It is okay if they eat candy now and then	31	3.0	31	3.0	31	2.9	35	3.0	27	2.9	34	3.0	31	3.0	30	2.9	33	3.0	31	3.0	32	3.0
If they do not like vegetables, I give them another nourishing thing	18	2.4	18	2.4	19	2.6	19	2.4	16	2.3	19	2.4	17	2.4	16	2.3	20	2.4	19	2.4	17	2.3
I give them industrial baked goods because that is what they like	11	2.5	12	2.5	14	2.4	10	2.5	12	2.5	11	2.5	13	2.5	9	2.4	12	2.5	12	2.5	10	2.5
At this age, salt is not harmful	10	2.3	10	2.3	5	2.1	10	2.2	7	2.2	11	2.3	9	2.3	9	2.3	11	2.3	9	2.2	11	2.3
Sugary soft drinks are good because they provide energy	9	2.3	9	2.3	12	2.2	8	2.3	9	2.3	10	2.3	10	2.3	9	2.3	10	2.4	9	2.3	10	2.3
It is better to eat what they want than to eat little	9	2.1	9	2.1	12	2.2	6	2.0	11	2.1	8	2.1	9	2.1	9	2.1	9	2.1	10	2.1	8	2.1
Since they are no longer a baby, there is no need to control their diet	6	1.8	6	1.8	9	1.9	8	1.8	6	1.8	7	2.0	5	1.7	7	1.8	8	1.9	6	1.8	7	1.8
If a child is chubby, it means they are healthy	5	2.0	5	2.0	9	2.1	6	1.9	6	2.0	5	2.0	7	2.0	5	2.0	3	1.9	7	2.0	4	1.9
Fruit is not necessary if I give them vitamins	4	1.7	4	1.7	3	1.8	4	1.7	5	1.7	3	1.6	3	1.6	5	1.7	4	1.7	4	1.7	4	1.7

**Table 4:** Percentage and averages of hygiene and health practices in families with children aged 6 to 14, according to household profile (nuclear, reconstituted, single parent), number of children, their age and their gender.

Hygiene and Health Practices	Total		Home Profile						Number of Children				Children’s Age						Children’s Gender			
	*n* = 1103		Nuclear *n* = 900		Reconstructed *n* = 59		Single Parent *n* = 144		Only Child *n* = 417		2 or More *n* = 686		6–8 *n* = 394		9–11 *n* = 351		12–14 *n* = 358		Boy *n* = 586		Girl *n* = 517	
	%	X	%	X	%	X	%	X	%	X	%	X	%	X	%	X	%	X	%	X	%	X
I make sure they sleep at least 8 h a day	93	4.4	94	4.4	88	4.3	89	4.4	93	4.4	93	4.4	94	4.5	93	4.4	92	4.3	92	4.4	94	4.4
They must have regular health check-ups with the paediatrician	86	4.2	86	4.2	83	4.3	84	4.2	87	4.2	85	4.2	89	4.3	87	4.2	81	4.1	86	4.2	85	4.2
Tooth brushing	89	4.9	90	4.9	80	4.9	82	4.9	89	4.9	87	4.9	88	4.9	87	4.9	88	4.9	89	4.9	88	4.9
Drinking a glass of milk daily	88	4.9	87	4.9	83	4.8	77	4.8	87	4.9	88	4.9	80	4.9	91	4.9	77	4.8	88	4.9	89	4.8
Daily shower or bath	89	4.8	88	4.8	87	4.8	88	4.8	80	4.8	82	4.8	89	4.8	87	4.8	85	4.8	85	4.8	87	4.8
Eating fish	70	3.7	65	3.8	58	3.5	60	3.7	66	3.8	60	3.7	80	3.8	76	3.7	73	3.7	77	3.7	78	3.7
Eating fast food (Mc Donald’s)	12	2.2	9	2.1	10	2.4	12	2.3	12	2.2	11	2.1	8	2.1	9	2.1	12	2.3	11	2.2	11	2.1
My kids go to the dentist at least once a year	80	4.1	81	4.1	80	4.1	73	3.9	77	4.1	82	4.1	78	4.1	83	4.2	80	4.1	80	4.1	81	4.1
When you have experience, you can give them medicine without going to the doctor, when you know what they have (cough, diarrhoea…)	9	1.9	9	1.8	10	2.0	11	2.0	9	1.8	9	1.9	7	1.8	10	1.9	10	1.9	9	1.9	9	1.8
